# Incremental Model Fit Assessment in the Case of Categorical Data: Tucker–Lewis Index for Item Response Theory Modeling

**DOI:** 10.1007/s11121-021-01253-4

**Published:** 2021-05-10

**Authors:** Li Cai, Seung Won Chung, Taehun Lee

**Affiliations:** 1 University of California, UCLA/CRESST, 315 GSEIS Bldg, Los Angeles, 90095-1522 CA USA; 2 University of Minnesota, Twin Cities, USA; 3 Chung-Ang University, Seoul, Korea

**Keywords:** Categorical data analysis, Model evaluation, Item response theory, Goodness of fit, Limited-information testing, TLI

## Abstract

The Tucker–Lewis index (TLI; Tucker & Lewis, 1973), also known as the non-normed fit index (NNFI; Bentler & Bonett, 1980), is one of the numerous incremental fit indices widely used in linear mean and covariance structure modeling, particularly in exploratory factor analysis, tools popular in prevention research. It augments information provided by other indices such as the root-mean-square error of approximation (RMSEA). In this paper, we develop and examine an analogous index for categorical item level data modeled with item response theory (IRT). The proposed Tucker–Lewis index for IRT (TLIRT) is based on Maydeu-Olivares and Joe's (2005) $M_2$ family of limited-information overall model fit statistics. The limited-information fit statistics have significantly better Chi-square approximation and power than traditional full-information Pearson or likelihood ratio statistics under realistic situations. Building on the incremental fit assessment principle, the TLIRT compares the fit of model under consideration along a spectrum of worst to best possible model fit scenarios. We examine the performance of the new index using simulated and empirical data. Results from a simulation study suggest that the new index behaves as theoretically expected, and it can offer additional insights about model fit not available from other sources. In addition, a more stringent cutoff value is perhaps needed than Hu and Bentler's (1999) traditional cutoff criterion with continuous variables. In the empirical data analysis, we use a data set from a measurement development project in support of cigarette smoking cessation research to illustrate the usefulness of the TLIRT. We noticed that had we only utilized the RMSEA index, we could have arrived at qualitatively different conclusions about model fit, depending on the choice of test statistics, an issue to which the TLIRT is relatively more immune.

**Supplementary Information:**

The online version of this article (10.1007/s11121-021-01253-4) contains supplementary material, which is available to authorized users.

## Introduction

Item response theory (IRT) is widely used in educational and psychological measurement research and practice (Thissen & Steinberg, 2009), and becoming more noticed in prevention research (e.g., Kirisci et al., 2001; Lac et al., 2016) as methods for the analysis of categorical data continue to develop. IRT models are nonlinear latent variable models for multivariate categorical data arranged in the form of multinomial contingency tables (Cai et al., 2016). Full-information maximum marginal likelihood (FIML) is the standard approach in IRT for parameter estimation (Wirth & Edwards, 2007).

As a prerequisite to any model-based statistical inference, and the subsequent policy or intervention decisions based on statistical analysis, evidence about model fit must be amassed. Just as a covariance structure model, an IRT model also seeks to represent population item response probabilities akin to a moment structure with a more parsimonious and interpretable set of parameters. Frameworks such as Cudeck and Henly (1991) that explicitly account for the presence of lack of model fit in the population are particularly helpful in this context.

The two most widely available IRT model fit statistics are Pearson’s $X^2$ statistic and the likelihood ratio statistic $G^2$ (Maydeu-Olivares, 2013). These test statistics depend on cell probabilities in the full item-by-item contingency table and hence are referred to as full-information test statistics. Despite their ease of computation, they have a serious drawback for testing IRT model fit, because the contingency tables for IRT modeling are often extremely sparse.

To solve this problem, limited-information goodness-of-fit tests e.g (Bartholomew & Leung, 2002; Cai et al., 2006; Maydeu-Olivares & Joe, 2006; Joe & Maydeu-Olivares, 2010) have emerged in the IRT literature. Maydeu-Olivares and Joe's (2005) test statistic, $M_2$, for example, uses residuals in the first- and second-order margins of the contingency table. These margins are better filled, and consequently, the test statistics have better calibration and power (Joe & Maydeu-Olivares, 2010). The chi-squaredness of these new test statistics also facilitates the derivation of fit indices that are sample size independent, such as the Root Mean Square Error of Approximation (RMSEA; Browne & Cudeck, 1993; Maydeu-Olivares & Joe, 2014). Additional $M_2$-inspired limited-information test statistics have been proposed by Cai and Hansen (2013) and Monroe and Cai (2015) for polytomous data.

The interpretation of RMSEA under categorical item level data, however, is not entirely without controversy. Standard cutoff values appear to be inadequate in accounting for potential differences caused by the number of categories (see Maydeu-Olivares & Joe, 2014; Monroe & Cai, 2015). In addition, while the proliferation of $M_2$-inspired test statistics have given researchers more choices, they further complicate the problem of interpreting RMSEA for categorical data. Each test statistic leads to a different estimate of the population non-centrality, a quantifier of the degree of model error in the population that is critical in computing the RMSEA. The degrees of freedom are also different across different test statistics. Taken together, Cai and Hansen (2013) and Monroe and Cai (2015) showed that RMSEA values computed from different flavors of $M_2$ statistic may lead to qualitatively different conclusions about model fit. We believe that while more research on RMSEA is much needed, alternative approaches should be considered.

We draw inspirations from limited-information estimation methods originating from the factor analysis tradition. These limited-information methods can be used to fit a restricted set of IRT models for ordinal data (see Forero & Maydeu-Olivares, 2009). Typically, a multistage weighted least-squares estimator is employed with intermediate moment matrices such as polychoric correlations. With these summary moment matrices, it is simple to specify a zero-factor model and obtain the relevant chi-squares for computing incremental fit indices such as the Tucker–Lewis index (TLI; Tucker & Lewis, 1973). Also known as the non-normed fit index (NNFI; Bentler & Bonett, 1980), TLI is one of the numerous incremental fit indices widely used in linear mean and covariance structure modeling, particularly in exploratory factor analysis. Because the TLI is based on the incremental fit assessment principle, we have reasons to believe that its application to IRT may be less affected by the number of categories and the choice of particular $M_2$ statistics, unlike the RMSEA (see Maydeu-Olivares & Joe, 2014); Monroe & Cai, 2015) for IRT models.

The only remaining issue is that FIML does not operate on the summary moment matrices, so we propose to solve the problem with limited-information goodness-of-fit statistics based on FIML parameter estimates. There are a number of reasons why this approach is the most practicable method in IRT research and data analytic practice. FIML is far more powerful in terms of its ability to handle complex data collection designs (e.g., with planned missing data, unequal probability of selection, and nesting relationships) as well as large data sets involving many hundreds of items and potentially many thousands of respondents. Wirth and Edwards (2007) discussed this latter point in detail. In addition, with FIML estimation, a much larger variety of IRT models, e.g., the so-called three-parameter logistic (3PL) model, nominal model, or polytomous models with adjacent-categories logit link functions become estimable. Such flexibility, while not necessarily deemed essential until recently for researchers who focus on data derived from Likert-type questionnaires, has always been mission-critical in a large segment of educational and psychological measurement. Even for Likert-type responses, more complex IRT models capable of handling additional construct-irrelevant facets of variation to improve the qualify of measurement routinely require FIML (e.g., Falk & Cai, 2016).

In the research presented here, we formalize and examine a new approach for incremental model fit assessment in IRT. The general recipe is as follows: use FIML to fit the IRT model, and then use a limited-information goodness-of-fit statistic to derive an incremental fit index with a null model implying full independence. This new index can be thought of as the IRT equivalent of the TLI out of classical structural equation modeling, especially exploratory factor analysis. These multivariate statistical tools are popular among prevention science researchers, so we will not belabor their importance or the TLI’s practical significance.

Our basic idea is deceptively simple: The fit of the IRT model under consideration is compared against that of a null model in a principled manner with a TLI-type index. The interpretation of the index leverages and modifies the standard guidelines for interpreting the TLI as widely understood and utilized in practice. The proposed Tucker–Lewis index for IRT (TLIRT) is based on the $M_2$ family of limited-information model fit statistics. In principle, any limited-information fit statistic with known asymptotic distribution properties may be used, e.g., Cai and Hansen' (2013) chi-square statistic, but we focus on Maydeu-Olivares and Joe's (2005) $M_2$ statistic, in particular. We will develop the index formally. Results from a set of simulation studies will be reported to examine the properties of the index to help gauge its interpretation in practice. Empirical data from a health-outcomes measurement development study in the smoking cessation research context will be used to illustrate the added value of the new index. It is important to note that all of the methods reported here are readily available for any substantive researcher’s use.

## A Brief Review of the Necessary Statistical Theory

### Model-building

To begin, let us consider an IRT model for dichotomous data. Let $U_i$ be a random variable indicating the response to item *i*, and let $u_i$ be a realization of $U_i$. In the case of dichotomously scored items, $U_i$ takes on two values, either 0 or 1. A plausible IRT model may be the multidimensional generalization of the two-parameter logistic (2PL) model, where the correct/endorsement/positive response (coded as $U_i = 1$) is modeled as a function of item parameters and latent variables:

1 $$\begin{aligned} P(U_i = 1 | \varvec{\eta }) = \frac{1}{1+\exp [-(\alpha _i+\varvec{\beta }_i'\varvec{\eta })]}, \end{aligned}$$

and $\alpha _i$ is the intercept term, $\varvec{\beta }_i$ a potentially vector-valued item slope parameter conformable with the dimensions of the latent variables $\varvec{\eta }$.

We use Samejima's (1969) graded response model (GRM) as an example of models for ordered categorical data. GRM for two categories is the 2PL model. For $K>2$ ordered categories, the GRM can be derived from the 2PL model. Upon defining the cumulative response probability for item *i* and category *k* according to a 2PL model

2 $$\begin{aligned} P (U_i \ge k | \varvec{\eta }) = \frac{1}{1+\exp [-(\alpha _{ik} + \varvec{\beta }_i'\varvec{\eta })]}, \end{aligned}$$

the category response probability is given by

3 $$\begin{aligned} P (U_i = k | \varvec{\eta }) = P (U_i \ge k | \varvec{\eta }) - P(U_i \ge k + 1 | \varvec{\eta }), \end{aligned}$$

for $k=0,...K-1$. Note that we choose to define $P (U_i \ge 0 | \varvec{\eta }) = 1$ and $P (U_i \ge K | \varvec{\eta }) = 0$ for the two boundary cases, so there are only $K-1$ intercept parameters.

Arguably the most important assumption in IRT modeling is the *conditional independence* assumption, which states that the conditional probability of a pattern of responses to *n* items factors into a product:

4 $$\begin{aligned} P\left( \bigcap _{i=1}^n U_i = u_i | \varvec{\eta }\right) = \prod _{i=1}^n P(U_i = u_i | \varvec{\eta }), \end{aligned}$$

were $\cap$ means the intersection of events. Equation (4) indicates that upon conditioning on (controlling for) the latent variable(s), the responses become independent. In other words, all observed correlatedness among item responses are presumed to be caused by the presence of $\varvec{\eta }$. This is not different from assumptions made in linear exploratory factor analysis about the lack of correlations among the unique factors.

Standard model-building approaches in IRT, (e.g., Bock & Aitkin, 1981) require the specification of a population (prior) distribution on the latent variable(s) in $\varvec{\eta }$. Upon introducing this prior $g(\varvec{\eta })$, the marginal response pattern probability becomes

5 $$\begin{aligned} P\left( \bigcap _{i=1}^n U_i = u_i \right) = \int \prod _{i=1}^n P(U_i = u_i | \varvec{\eta }) g(\varvec{\eta }) d\varvec{\eta }. \end{aligned}$$

In contrast to conditional independence, as embodied by Equations (4) and (5), a complete-independence model does not contain any latent variables at all

$$\begin{aligned} P\left( \bigcap _{i=1}^n U_i = u_i \right) = \prod _{i=1}^n P(U_i = u_i). \end{aligned}$$

It may be considered a worst possible case when the specification of factor analytic dimensions is of interest. Functionally, the complete-independence (zero-factor) model has item intercepts only and no item slopes.

### Parameter Estimation

Using conventional notation from statistical sciences, let us refer to all the free item parameters generically as $\varvec{\theta }$. The task of item calibration is to estimate $\varvec{\theta }$. For a sample of item response data from *N* respondents to *n* items, fitting an IRT model to this $N\times n$ categorical data matrix using a standard software package leads to the maximum likelihood estimate, standard errors, and log-likelihood statistics.

To elaborate, the IRT model’s likelihood is based on a multinominal distribution with $C=K^n$ cells. Upon knowing the item parameters, each cell probability is uniquely determined by the pattern of responses (the $u_i$’s). Therefore, we may conveniently sort all possible response patterns in the lexicographical ordering, e.g., from $(0,\ldots ,0)$ to $(1,\ldots ,1)$ for all dichotomous responses, and index them as $c = 1,\ldots ,C$. Consequently, we may write a generic marginal response pattern probability (Equation 5) as $\pi _c(\varvec{\theta })$, where the parentheses emphasize the dependence of $\pi _c$ on the item parameters. The IRT model is said to be correctly specified if there exists some $\varvec{\theta }_0$ such that $\pi _c(\varvec{\theta }_0) = \pi _{0c}$, for all *c*, where $\pi _{0c}$ may be referred to as the true multinomial proportions.

Correspondingly, the observed item response data can be rearranged into response patterns by proportions, i.e., in grouped format as an *n*-way contingency table. Given a random sample of *N* respondents, we shall use $p_c$ to denote the observed proportion of individuals that have response pattern *c*. We see that the IRT model is directly defined on a multinomial with *C* cells. The likelihood function is

6 $$\begin{aligned} L(\varvec{\theta }) \propto \prod _{c=1}^C [\pi _c(\varvec{\theta })]^{p_c}. \end{aligned}$$

Maximization of $L(\varvec{\theta })$ leads to the maximum marginal likelihood estimate of the item parameters $\varvec{\hat{\theta }}$. A standard approach is FIML with the EM algorithm (e.g., Bock & Aitkin, 1981), which we use throughout.

Equivalently, one may also choose to minimize the following likelihood ratio discrepancy function

7 $$\begin{aligned} G(\varvec{\theta }) = 2 \sum _{c=1}^C p_c \log \left( \frac{p_c}{\pi _c(\varvec{\theta })}\right) . \end{aligned}$$

Under the null hypothesis of exactly correct model specification, the minimum discrepancy function $G(\varvec{\hat{\theta }})$, when multiplied by *N*, is distributed as a central chi-square random variable with $C-1-\dim (\varvec{\theta })$ degrees-of-freedom when *N* is several times of *C*. *N* times $G(\varvec{\hat{\theta }})$ is also referred to as the likelihood ratio $G^2$ test statistic in categorical data analysis:

8 $$\begin{aligned} G^2 = 2 N \sum _{c=1}^C p_c \log \left( \frac{p_c}{\hat{\pi }_c}\right) , \end{aligned}$$

where $\hat{\pi }_c = \pi _c(\varvec{\hat{\theta }})$ is the model-implied response probability under maximum likelihood estimation. As an alternative to $G^2$, the asymptotically equivalent Pearson $X^2$ statistic may be employed for overall model fit testing as well:

9 $$\begin{aligned} X^2 = N \sum _{c=1}^C \frac{(p_c - \hat{\pi }_c)^2}{\hat{\pi }_c}. \end{aligned}$$

Note that $p_c - \hat{\pi }_c$ is the residual from cell *c* of the underlying item-by-item contingency table.

### Limited-information Model Fit Testing

As mentioned earlier, both $G^2$ and $X^2$ statistics require large cell frequencies for the chi-square approximation to work. This is rarely if ever true in the practice of IRT modeling (Bartholomew & Tzamourani, 1999), where the length of the tests are often long and the value of *C* astronomically large, making the contingency table extremely sparse. Under sparseness, the Type I error rates of $G^2$ and $X^2$ become severely mis-calibrated. As sparseness becomes more severe, the power of $G^2$ to detect model misspecification plummets, while that of $X^2$ becomes far too elevated to be useful as all models are rejected (see e.g., Cai et al., 2006). The practical utility of $G^2$ and $X^2$ is dubious in IRT.

As a solution to the problem of sparseness, psychometric researchers have turned increasingly to limited-information goodness-of-fit test statistics that are based on marginal residuals (Maydeu-Olivares & Joe, 2005, 2006; Cai et al., 2006; Joe & Maydeu-Olivares, 2010; Maydeu-Olivares & Montaño, 2012). These test statistics can maintain adequate Type I error rates even when the contingency table is sparse, and they can be more powerful than the full-information counterparts.

In the theory developed by Maydeu-Olivares and Joe (2005) marginal residuals up to order 2 are used as opposed to the multinomial cell residuals. For *n* dichotomous items, there are *n* first-order marginal residuals, and $n(n-1)/2$ second-order marginal residuals. Let them be denoted as $\mathbf {e}_2$. The $M_2$ statistic is defined as

10 $$\begin{aligned} M_2 = N\mathbf {e}'_{2} \left[ \varvec{\hat{\Xi }}_2^{-1} - \varvec{\hat{\Xi }}_2^{-1}\varvec{\hat{\Delta }}_2{\left( \varvec{\hat{\Delta }}_2'\varvec{\hat{\Xi }}_2^{-1}\varvec{\hat{\Delta }}_2\right) }^{-1}\varvec{\hat{\Delta }}_2'\varvec{\hat{\Xi }}_2^{-1}\right] \mathbf {e}'_{2}, \end{aligned}$$

where $\varvec{\hat{\Xi }}_2$ represents an error covariance matrix for the marginal probabilities, and $\varvec{\hat{\Delta }}_2$ represents the Jacobian matrix associated with the marginal probabilities. The details are fully described in the online supplemental material. It suffices to state that $M_2$ is asymptotically chi-square distributed with $n(n+1)/2 - \dim (\varvec{\theta })$ degrees of freedom under the null hypothesis that the model fits *exactly* in the population. Furthermore, under the alternative hypothesis (lack of fit), the statistic will behave like a non-central chi-square variate when *N* is large (Browne, 1984).

## Tucker–Lewis Index for IRT Models

All statistical models, IRT models included, have one thing in common: *They are all wrong to some degree* (Box, 1979; MacCallum, 2003). The notion of model being always imperfect implies that even if a proposed IRT model is closely approximating the structure of constructs being measured, that model will not exactly reproduce the response pattern probabilities in the population. This general lack of fit of a model in the population has been known as *model error* (MacCallum & Tucker, 1991), *approximation discrepancy* (Cudeck & Henly, 1991) or a *fundamental contradiction* between the model and the real world (Meehl, 1990). Recognizing such imperfection of statistical models, there is little to be gained by testing the hypothesis that a postulated IRT model holds *exactly* in the population, which is never true. A statistically significant result merely suggests that the researchers has adequate sample size to reject the null hypothesis of exact fit.

In the context of factor analysis and structural equation models, discussions about the problems associated with testing hypotheses that are never true date back to Thurstone (1930, p.469), who used the phrase “practical irrelevancies” to represent the aspects of real world that cause violations of a model but might well be of little practical importance to the meaningfulness or utility of the model (MacCallum, 2003, p.115). Beyond simple acknowledgment of imperfection of statistical models, Tucker and Lewis (1973) proposed a practical index for factor analysis and structural equation models. Specifically, TLI evaluates the incremental improvement in fit of a given substantive model over that of a null model, which corresponds to one of the worst possible representations of the data (Widaman & Thompson, 2003). To quantify the degree of improvement, the substantive model’s incremental improvement in fit is compared against that of an ideal model in the form of a ratio.

We follow the same principle and propose a TLI for evaluating incremental model fit in IRT. As in factor analysis and structural equation modeling (SEM), a zero-factor or complete-independence model can serve as the null model. This choice of the null model is of course not without its own fair share of controversies, as is true of many discussions in model fit assessment. Widaman and Thompson (2003) were among the first to raise general concerns about the choice of the null model in incremental fit assessment. A “no common factor” null model is a useful starting point for discussions about the TLIRT because it is strongly tied to latent dimensionality specification. The complete-independence model is a plausible but not the only plausible model. In the case of testing a sequence of progressively constrained models, e.g., the difference between the fit of a 2PL IRT model and a Rasch model, the most constrained model may well serve as a more viable null model, provided that the test statistic chosen as the basis of TLIRT has sufficient power to discern the differences in fit. If, on the other hand, the main interest is on dimensionality specification, which often precedes more detailed work on item model fit, the zero-factor null model offers some important insights that should be examined first.

Instead of $X^2$ or $G^2$ statistics, we propose to use $M_2$-type statistics to take advantage of the more superior chi-square approximation. Specifically, the Tuker-Lewis Index for IRT models, termed TLIRT hereafter can be computed as

11 $$\begin{aligned} \text {TLIRT} = \frac{\chi ^2_0 / \texttt {df}_0 - \chi ^2_{m} / \texttt {df}_m}{\chi ^2_0 / \texttt {df}_0 -1}, \end{aligned}$$

where $\chi ^2_0$ and $\chi ^2_m$ represents the observed values of $M_2$ for the null model and the substantive model being evaluated, respectively. The $\texttt {df}_0$ and $\texttt {df}_m$ represents their respective degrees of freedom. The chi-square to degrees of freedom ratios provide the per-degree-of-freedom lack of fit and one can see that they estimate the ratios in the population definition. For the null model, the ratio is expected to be large. For an ideal model that fits well, the ratio is expected to be one. Therefore, the numerator represents the substantive model’s incremental fit improvement over the null model and the denominator represents the ideal model’s improvement over the null. We note that the sample value of TLIRT may be outside of the (0,1) interval but a higher value is generally indicative of better model fit.

## Simulation Study

A simulation study was conducted to evaluate the performance of TLIRT. Specifically, we investigate how TLIRT based on the $M_2$ family of fit statistics behaves for unidimensional IRT model under correct dimensionality specification and misspecification. We examine the effectiveness of conventional TLI cutoff values for IRT models. Four sample sizes ($N=500, 1000, 2500, 5000$) and two conditions for the number of categories per item ($K=2, 5$) were considered. The length of the simulated test is 30 items.

The generating item model is the GRM. In order to systematically vary the item location and discrimination parameters, we adopted an alternative parameterization of the item intercepts akin to Muraki's (1990) rating scale formulation. Recall in the case of unidimensional IRT, where $\eta$ is a scalar, the linear predictor in the graded model may be equivalently expressed as $\alpha _{ik} + \beta _i\eta = \beta _i (\eta - b_{i} + d_{k})$, where $b_{i}$ is an overall location parameter for the item, and the set of deviation (step) parameters $d_{k}$ describe category boundaries. For identification the sum of $d_{k}$’s should be 0. In the case of $K=2$, the step term vanishes. It is clear that $\alpha _{ik} = -\beta _i(b_i-d_{k}) = -\beta _i b_{ik}$, where $b_{ik} = b_i - d_{k}$ is the traditional graded difficulty parameter.

The location and step parameterization is easier to systematically manipulate and allows us to cover plausible regions of the parameter space more easily. In our study, we first considered three different realistic values for the slope parameter: 1.0, 1.5, and 2.0. Next, we chose realistic values for the overall item location parameter ranging from -1.0 to 1.0 with equal spacing of 0.5. We then matched each slope parameter with every item location parameter such that poorly and highly discriminating items are matched with all types of item difficulty. We then chose a set of step parameters that are roughly equally spaced within an item: for $K = 5$, the step parameters are $(1.2, 0.4, -0.4, -1.2)$. This creates the parameter values for the first 15 items (items 1 - 15). We duplicated the same parameter values for the remaining 15 items (items 16 - 30). The generating parameter values are displayed in Table 1.

**Table 1:** Generating Parameter Values

		$K=2$	$K=5$							
Items	$\beta _i$	$b_{i1}$	$d_1$	$d_2$	$d_3$	$d_4$	$b_{i1}$	$b_{i2}$	$b_{i3}$	$b_{i4}$
1, 16	1.0	-1.0	1.2	0.4	-0.4	-1.2	-2.2	-1.4	-0.6	0.2
2, 17	1.5	-1.0	1.2	0.4	-0.4	-1.2	-2.2	-1.4	-0.6	0.2
3, 18	2.0	-1.0	1.2	0.4	-0.4	-1.2	-2.2	-1.4	-0.6	0.2
4, 19	1.0	-0.5	1.2	0.4	-0.4	-1.2	-1.7	-0.9	-0.1	0.7
5, 20	1.5	-0.5	1.2	0.4	-0.4	-1.2	-1.7	-0.9	-0.1	0.7
6, 21	2.0	-0.5	1.2	0.4	-0.4	-1.2	-1.7	-0.9	-0.1	0.7
7, 22	1.0	0.0	1.2	0.4	-0.4	-1.2	-1.2	-0.4	0.4	1.2
8, 23	1.5	0.0	1.2	0.4	-0.4	-1.2	-1.2	-0.4	0.4	1.2
9, 24	2.0	0.0	1.2	0.4	-0.4	-1.2	-1.2	-0.4	0.4	1.2
10, 25	1.0	0.5	1.2	0.4	-0.4	-1.2	-0.7	0.1	0.9	1.7
11, 26	1.5	0.5	1.2	0.4	-0.4	-1.2	-0.7	0.1	0.9	1.7
12, 27	2.0	0.5	1.2	0.4	-0.4	-1.2	-0.7	0.1	0.9	1.7
13, 28	1.0	1.0	1.2	0.4	-0.4	-1.2	-0.2	0.6	1.4	2.2
14, 29	1.5	1.0	1.2	0.4	-0.4	-1.2	-0.2	0.6	1.4	2.2
15, 30	2.0	1.0	1.2	0.4	-0.4	-1.2	-0.2	0.6	1.4	2.2

To simulate model misspecification, we allowed minor degrees of misspecification in terms of unmodeled dimensions. The data generating model is a 2-dimensional model with independent cluster factor pattern, but the fitted model is unidimensional. For the generating model, the first 15 items load on the first dimension and the remainder load on the second factor. The latent correlation between the two dimensions is .8, which is a high value but it does not imply that the dimensions are the same. Note that we consider such a factor structure to investigate whether TLIRT is capable of detecting the misspecification, which is routinely needed in operational and research settings.

To home in on the viability of conventional TLI cutoff values, a more realistic data generating condition was also investigated. Specifically, the Tucker et al. (1969) procedure was applied to introduce model error. Tucker et al. (1969) described three types of latent variables in a factor analytic framework: major domain factors, minor factors, and unique factors. They explicitly postulated that minor factors may influence more than one observed variables and introduce extra correlations among the observed variables that cannot be parsimoniously accounted for by the inclusion of major domain factors alone.

The TKL procedure has been used in settings such as common factor analysis (e.g., MacCallum & Tucker, 1991) and multidimensional IRT (e.g., Cai & Hansen, 2013). Using the relationship between categorical factor analysis model and item response model (Wirth & Edwards, 2007), the procedure begins with the transformation of the generating slope parameters to factor loadings. In the current implementation of the TKL procedure, the factor loadings for the minor factors are generated randomly with a mean of 0 but has progressively decreasing variability such that successive columns for the minor factor loading matrix have standard deviations equal to 80% of the preceding columns. It is followed by a scaling procedure so that each item’s variance becomes unity. The minor factor loadings generated in this manner introduce a more pervasive kind of model error due to unmodeled dimensionality that cannot be parsimoniously represented by any conventional IRT model. After the TKL procedure is performed, the factor loadings for major and minor factors are transformed back to the corresponding slope parameters. Here, we specified 2 major domain factors (i.e., a 2-dimensional model) with 50 minor factors. Thus a total of 52 slope parameters were generated for each item. The degree of contribution from the minor factors to the total variance was set to 10%, reflecting a moderate degree of model error that can be found with real data.

To summarize, we have three data generating models in the simulation study: 1) the Null condition, where the generating model is unidimensional, 2) Misspecification I, where the generating model is a substantially (.80) correlated 2-dimensional model, and 3) Misspecification II, where the generating model has 2 major domain factors and 50 minor factors introduced by the TKL procedure. The fitted model remains unidimensional throughout. The total number of replications in each condition was set to 500. We used the R software (R Core Team, 2017) for data generation and flexMIRT® (Cai, 2015) for model fitting. The $M_2$ statistic values were collected from the software package flexMIRT® (Cai, 2015) output. We tabulated the TLI values and their confidence intervals. We also obtained RMSEA values to serve as a point of reference.

### Results Under the Null Condition

Table 2 presents the means, variances, empirical rejection rates at .01, .05 and .10 alpha levels of, as well as the means and empirical 90% confidence intervals of RMSEA and TLIRT, under the null condition for $M_2$. Under our simulation design, $M_2$ seems to be well behaved. It approximately follow the purported chi-square distribution, and the empirical rejection rates closely match the nominal levels. In addition, there appears to be no appreciable difference across the number of categories and sample size. For all conditions, the mean RMSEA values are small, ranging from .000 to .005, and the mean TLIRT value is consistently equal to 1.000. This is as expected. RMSEA indicates that the population non-centrality is at or close to zero, and the TLIRT indicates the the model is at or close to ideal fit. One should only interpret this as a confirmation of our statistical theory and implementation because the empirical data are never this clean.

**Table 2:** Simulation Results: The Null Condition

	$M_2$			Rejection Rates			RMSEA		TLIRT	
*K*	*N*	Mean	Var	.01	.05	.1	Mean	90% CI	Mean	90% CI
2	500	405.9	865.77	.012	.072	.114	.005	(.000, .020)	1.000	(1.00, 1.00)
	1000	406.4	804.299	.020	.052	.092	.004	(.000, .010)	1.000	(1.00, 1.00)
	2500	403.5	765.629	.006	.032	.074	.002	(.000, .010)	1.000	(1.00, 1.00)
	5000	404.5	807.042	.012	.048	.102	.000	(.000, .000)	1.000	(1.00, 1.00)
5	500	6942.1	15307.4	.024	.068	.132	.003	(.000, .010)	1.000	(.99, 1.01)
	1000	6936.9	13418.9	.014	.056	.090	.001	(.000, .010)	1.000	(.99, 1.00)
	2500	6930.3	13116.9	.012	.036	.090	.000	(.000, .000)	1.000	(1.00, 1.00)
	5000	6928.2	15711.0	.010	.054	.114	.000	(.000, .000)	1.000	(1.00, 1.00)

*K*: number of categories; *N*: sample size; When $K=2$, degrees-of-freedom is 405. When $K=5$, degrees-of-freedom is 6930

### Results Under Misspecification

Next, we present in Tables 3 and 4 the results under model misspecification using a similar format as Table 2. Recall that Misspecification I and II both represent dimensionality misspecifications. The latter condition has an extra layer of model error introduced with the TKL procedure.

**Table 3:** Simulation Results: Model Misspecification I

		$M_2$		Rejection Rates			RMSEA		TLIRT	
*K*	*N*	*df*	Mean	0.01	0.05	0.1	Mean	90% CI	Mean	90% CI
2	500	405	699.1	1.00	1.00	1.00	.038	(.030, .040)	.968	(.96, .98)
	1000	405	926.0	1.00	1.00	1.00	.036	(.030, .040)	.977	(.97, .98)
	2500	405	1732.3	1.00	1.00	1.00	.038	(.030, .040)	.975	(.97, .98)
	5000	405	3024.7	1.00	1.00	1.00	.038	(.030, .040)	.974	(.97, .98)
5	500	6930	7779.8	1.00	1.00	1.00	.017	(.010, .020)	.961	(.95, .97)
	1000	6930	8356.7	1.00	1.00	1.00	.013	(.010, .020)	.973	(.97, .98)
	2500	6930	10609	1.00	1.00	1.00	.012	(.010, .020)	.970	(.97, .97)
	5000	6930	14334	1.00	1.00	1.00	.012	(.010, .010)	.970	(.97, .97)

*K*: number of categories; *N*: sample size

**Table 4:** Simulation Results: Model Misspecification II

		$M_2$		Rejection Rates			RMSEA		TLIRT	
*K*	*N*	*df*	Mean	0.01	0.05	0.1	Mean	90% CI	Mean	90% CI
2	500	405	695.4	1.00	1.00	1.00	.038	(.030, .040)	.972	(.97, .98)
	1000	405	1012.3	1.00	1.00	1.00	.039	(.030, .040)	.973	(.97, .98)
	2500	405	1934.5	1.00	1.00	1.00	.040	(.040, .040)	.970	(.97, .97)
	5000	405	3490.2	1.00	1.00	1.00	.040	(.040, .040)	.970	(.97, .97)
5	500	6930	7751.2	1.00	1.00	1.00	.016	(.010, .020)	.966	(.96, .98)
	1000	6930	8613.9	1.00	1.00	1.00	.017	(.010, .020)	.965	(.96, .97)
	2500	6930	11012.6	1.00	1.00	1.00	.018	(.010, .020)	.965	(.96, .97)
	5000	6930	15815.2	1.00	1.00	1.00	.020	(.020, .020)	.962	(.96, .97)

*K*: number of categories; *N*: sample size

The results of Misspecification I and Misspecification II are comparable. The empirical rejections rates are 1.0 across all conditions, which suggests that $M_2$ is very powerful in detecting even mild dimensionality misspecification. This is consistent with findings from the literature. The TLIRT values range from .961 to .977 under Misspecification I and .962 to .973 under Misspecification II. We will shortly further examine whether this affects the choice of TLIRT cutoff values.

Our major concern is the performance of TLIRT, particularly in comparison to RMSEA. As expected from prior research (e.g., Cai & Hansen, 2013; Maydeu-Olivares & Joe, 2014; Monroe & Cai, 2015), RMSEA values vary with the number of categories in both misspecification conditions. Specifically, the mean RMSEA values is significantly lower for $K=5$ than for $K=2$. The values range from .012 to .020 for $K=5$ and from .036 to .040 for $K=2$. The noncentrality parameter, key to the computation of RMSEA, appears to increase more slowly with the increase in the number of categories. Turning to TLIRT, it appears that under Misspecification I, the number of categories made barely if any difference. For Misspecification II, the overall value of TLIRT decreased only slightly (in the second decimal), with largely comparable confidence intervals whether *K* is 2 or 5.

Finally, we calculated the rejection rates for the two misspecified models at different levels of TLI cutoff values. Table 5 displays the rejection rates under TLIRT=.96, .97, .98 and .99 for Misspecification I and Misspecifiaiton II. With a cutoff value of .96, the misspecified models were rarely rejected, ranging from 0% to 19.4% but mostly 0%. With a cutoff value of .97, TLIRT rejected 27.6% and 69.8% misspecified model I for $K=2$ and $K=5$, respectively when $N = 500$. With the same cutoff value, TLIRT rejected 40.6% to 78.2% misspecified model II for $K=5$. With a cutoff value of .98, TLIRT rejected 34.6% to 85.4% and 65.8% to 100% of the misspecified model I for $K=2$ and $K=5$, respectively, and 67.2% to 98.8% and 91.8% to 100% of the misspecified model II for $K=2$ and $K=5$, respectively. Clearly, higher rejection rates were observed for $K=5$. With a cutoff value of .99, we observe that both misspecified models were rejected at about 100%.

**Table 5:** Simulation Results: Rejection Rates (%) at Cutoff Values

			Cutoff value			
	*K*	*N*	.96	.97	.98	.99
Misspecification I	2	500	3.6	27.6	85.4	99.4
		1000	0.0	0.0	34.6	99.6
		2500	0.0	0.0	47.6	100.0
		5000	0.0	0.0	61.4	100.0
	5	500	19.4	69.8	96.2	100.0
		1000	0.0	2.0	65.8	100.0
		2500	0.0	1.8	99.2	100.0
		5000	0.0	0.8	100.0	100.0
Misspecification II	2	500	0.2	11.4	67.2	99.2
		1000	0.0	2.6	70.8	100.0
		2500	0.0	1.2	96.0	100.0
		5000	0.0	0.0	98.8	100.0
	5	500	4.2	42.8	91.8	100.0
		1000	1.0	48.6	99.6	100.0
		2500	0.0	40.6	100.0	100.0
		5000	0.0	78.2	100.0	100.0

*K*: number of categories; *N*: sample size

The simulation study finds that the proposed TLIRT index tends to be somewhat higher than what is typically observed for standard linear covariance structure modeling. Consequently, reasonable cutoff values may be well above the conventional cutoff point employed in the factor analysis literature (Hu & Bentler, 1999). Overall, we conjecture that a reasonable cutoff should be above .97, but more research is clearly needed.

## Empirical Example

In this section, we compare the proposed TLIRT index based on the $M_2$ statistic from FIML estimation, and the much more familiar TLI based on robust test statistics from more conventional limited-information estimation procedurs, i.e., diagonally weighted least square (DWLS) and unweighted least square (ULS) implemented in structural equation modeling. Specifically, the test statistics with first- and second-order corrections using a scale-shift approach (Asparouhov & Muthem, 2010) were used. Note that TLI or RMSEA values using the limited-information method are easily obtained from existing SEM software packages. We used the *lavaan* package in (R Core Team, 2017) to fit the model and compute the fit indices using limited-information methods.

This empirical data came from the Patient-Reported Outcomes Measurement Information System (PROMIS). Specifically, we utilized data from the PROMIS Smoking Initiative (Edelen et al., 2012), a measurement development and validation project in the context of smoking cessation research. A total of 277 smoking items were administered to a sample of daily and non-daily smokers. Before one can begin to provide scores on constructs measured by these items or use the item bank for computerized adaptive tests, exploration and confirmation of the underlying dimensionality of the item pool is necessary. To minimize respondent burden, blocks of items were constructed so that each respondent was administered two blocks randomly (see Edelen et al., 2012; Hansen et al., 2014). This creates a planned missing completely at random design. The item ratings were on a 5-point ordinal scale (e.g., not at all, a little bit, somewhat, quite a bit, very much). Based on statistical analysis and content review, researchers concluded that six major domains were present: nicotine dependence, coping expectancies, emotional and sensory expectancies, health expectancies, psychosocial expectancies, and social motivations. Example items for each domain are presented in Table 6.

**Table 6:** Examples Items for PROMIS Smoking Initiative

Construct	Example Items
Nicotine Dependence	My desire to smoke seems overpowering.
Coping Expectancies	I rely on smoking to deal with stress.
Emotional and Sensory Expectancies	I enjoy the steps I take to light up a cigarette.
Health Expectancies	Smoking is taking years off my life.
Psychosocial Expectancies	I feel embarrassed when I smoke.
Social Motivations	I enjoy the social aspect of smoking with other smokers.

Here we only used data from daily smokers ($N = 4,201$) and randomly selected 5 items from the 6 domains, yielding 30 items in total. We down-sampled items because we would not want to overburden the limited-information methods. As Wirth and Edwards (2007) noted, limited-information methods tend to work well when the number of items is not large. The data set comes with a substantial amount of missing data due to design. The fitted model is a bifactor/testlet multidimensional IRT model having one general factor and 6 domain-specific factors. Within-item proportionality constraints representing testlets were specified. We obtained $M_2$ from flexMIRT ® (Cai, 2015), which is 5544.30 ($df=6924, p<0.001$), and the RMSEA based on $M_2$ is .00 with a 90% confidence interval of [0, .003] and the TLIRT value is 1.00. The RMSEA and TLIRT values suggest the model fits the data well. We are not surprised by this finding because it confirms a substantial amount of results and analyses reported earlier (see, e.g., Hansen et al., 2014) about the dimensionality structure of this instrument.

However, the corresponding indices using the limited-information method could not be obtained from the *lavaan* package with the default setting (listwise deletion). This is because we are left with no observations, if limited-information estimation coupled with listwise deletion is employed, due to the presence of missing values in every variable due to the planned missing data design. When pairwise deletion was used, $T_{DWLS}=8137.86$ ($df=405, p<.001$), and the RMSEA is .072 with a 90% confidence interval of [.071, .073] while the reported TLI value is .707. In addition, $T_{ULS}=6816.11$ ($df=405, p<0.001$), and the RMSEA is .061 with a 90% confidence interval of [.060, .063], with a corresponding TLI value of .602. Note that $T_{DWLS}$ and $T_{ULS}$ are the robust test statistics with the first- and second-order corrections computed from DWLS and ULS estimates, respectively. The result may be surprising because it seems to be inconsistent with the IRT-based results reported earlier. Here, we should remind ourselves of Enders and Bandalos (2001) comments on potentially erroneous statistical inference from pairwise deletion under missing data.

In case one wonders about the unique nature of planned missing data. We now use a subset of data from the same study that do not contain any missing values. This enables the comparison of TLI values from an analogous categorical factor analysis using the limited information method to TLIRT. There are 12 items in the PROMIS smoking study that attempt to measure positive consequences of nicotine (PCN; see Table 7). The total sample size is $N = 2,717$ for this subset.

**Table 7:** Item Stems for PCN Items

Item	Stem
1.	Smoking helps me concentrate.
2.	Smoking helps me think more clearly.
3.	Smoking helps me stay focused.
4.	Smoking makes me feel better in social situations.
5.	Smoking makes me feel more self-confident with others.
6.	Smoking helps me feel more relaxed when I’m with other people.
7.	Smoking helps me deal with anxiety.
8.	Smoking calms me down.
9.	If I’m feeling irritable, a cigarette will help me relax.
10.	Smoking a cigarette energizes me.
11.	Smoking makes me feel less tired.
12.	Smoking perks me up.

Items 1-3 and 10-12 represent an arousal aspect of smoking, and items 4-9 imply a calming aspect of smoking, and hence a two-factor structure may be plausible. Accordingly, we fitted a GRM with 2 correlated factors to the data. The test statistics and their RMSEA and TLIRT values from flexMIRT and the *lavaan* package are presented in Table 8.

**Table 8:** TLI for PCN items from various statistics

	Statistic			RMSEA		TLIRT
Test statistic	Value	*df*	*p*	Value	90% CI	Value
$M_2$	7800.17	1031	$<.001$	.05	(.049, .051)	.92
$T_{DWLS}$	3665.30	53	$<.001$	.16	(.154, .163)	.94
$T_{ULS}$	3328.79	53	$<.001$	.15	(.147, .155)	.91

Similar TLIRT values were observed, with the values from $M_2$, $T_{DWLS}$ and $T_{ULS}$ being .92, .94, and .91, respectively. Interestingly, the value from $M_2$ lies between values from the two limited-information methods. Using the cutoff suggested in our simulation study, all values are below .97, and hence the model does not provide a good fit to the data. The qualitative conclusions based on TLI do not differ regardless of whether full or limited-information methods were employed.

On the other hand, the RMSEA computed from $M_2$ differs considerably from those based on $T_{DWLS}$ or $T_{ULS}$. RMSEA derived from $M_2$ is .05 with a 90% confidence interval of [.049, .051], and those from $T_{DWLS}$ and $T_{ULS}$ are .16 with a 90% confidence interval of [.154, .163] and .15 with a 90% confidence interval of [.147, .155], respectively. If we follow the standard guidelines for interpreting RMSEA in factor analysis or SEM (Browne & Cudeck, 1993), we come to the conclusion of either “close fit,” if we use the RMSEA based on $M_2$, or “unacceptable fit,” if we use RMSEA based on $T_{DWLS}$ and $T_{ULS}$. Of course, existing literature on IRT-based RMSEA with categorical data (e.g., Maydeu-Olivares & Joe, 2014) already indicates that a potentially different (and likely more stringent) cutoff value may be warranted, so the qualitative conclusions about the lack of model fit might be similar in this case. The difference in RMSEA magnitude across FIML and limited-information estimation methods, however, is noticeable.

Up to this point, $M_2$, and its derived RMSEA provide the main indices for gauging IRT model fit under FIML estimation. The proposed TLIRT and our preliminary investigations indicate that TLIRT offers added information about an important aspect of model fit. With this new knowledge, we conclude that the two-factor GRM does not fit the data adequately.

## Discussions

We introduced a new comparative model fit index for IRT modeling, the TLIRT. The proposed index is based on FIML estimation, followed by limited-information goodness-of-fit testing. Both approaches have become routine in the IRT literature. FIML is often more desirable, especially with the complexities of real research design in educational and psychological measurement, such as when planned missing data are present or more sophisticated item models may be required. When both limited-information and full-information methods may be applicable, our newly proposed TLIRT index appear to perform similarly as statistics from limited-information methods.

Our simulations showed that a more stringent cutoff criterion than the conventional TLI cutoff in linear mean and covariance structure modeling should be applied for categorical data. We generated minor but noticeable and substantively meaningful model misspecifications. We noted that a TLIRT value of around .97 to .98 seems to be a reasonable range insofar as general guidance on cutoff values may be needed. It should be noted, however, we strongly recommend against using the suggested cutoff criterion as a one-size-fits-all rule. It is only useful to the extent that the type of misspecification is consistent with the assumption made in the simulation, i.e., mild dimensionality misspecification. To understand and interpreting the TLIRT well, more research is needed. For instance, confidence interval for TLIRT could be derived and it may provide additional information to researchers.

In addition to the type of misspecification, we see that the number of categories and the type of statistics may require further examination. Our speculation is that the TLIRT does not depend so heavily on the number of categories and model size (largely driven by test length) as the RMSEA because it avoids the complicated interactions between noncentrality and degrees of freedom. This positive aspect of the TLIRT should be further investigated in future research. The sensitivity to the choice of limited-information model fit statistics such as Cai and Hansen (2013) and Monroe and Cai (2015) is another possibly avenue of future research. We also recommend a “two-index presentation strategy” as noted by Hu and Bentler (1999) and among others.

Another commonly used model fit index in the context of factor analysis and SEM is the comparative fit index (CFI; Bentler, 1990). Its adaptation to IRT modeling is certainly possible. However, we opted for TLI since it was formulated with a strong correction for model complexity stemming from its “mean-square” approach. On the other hand, CFI does little correction or adjustment for model complexity because it is based on a “sums-of-squares” metric. It is speculated that the interpretation of the IRT version of CFI would not be the same as the CFI in SEM, akin to TLIRT. Nevertheless, its exact behavior needs to be studied further as the theory behind the two indices are different.

In sum, initial evidence leads us to believe that TLIRT is a promising index to aid the evaluation of IRT models. As a final remark, Widaman and Thompson's (2003) general concern about the choice of the null model in incremental fit indices are still valid in future discussions of TLIRT. Much is still incumbent on the researcher to articulate what represents a plausible worst-case model in any given measurement situation. Some interesting special cases may have to be developed for multiple-group IRT models widely used in scale alignment and differential item functioning research. The research reported here may be one small step toward better IRT model appraisal in prevention science.

## Supplementary Information

Below is the link to the electronic supplementary material.

Supplementary file1 (DOC 102 kb)
