# Supplementary material for: Incremental Model Fit Assessment in the Case of Categorical Data: Tucker–Lewis Index for Item Response Theory Modeling
Source: Prev Sci. 2021 May 10;24(3):455–66. doi: 10.1007/s11121-021-01253-4 (PMC10115722; doi:10.1007/s11121-021-01253-4)
Supplement: Supplementary file 1 — Supplementary file1 (DOC 102 kb) [file 11121_2021_1253_MOESM1_ESM.pdf]

Online Appendix to *Incremental Model Fit Assessment in the Case of Categorical Data: Tucker-Lewis Index for Item Response Theory Modeling* by Cai, Chung, and Lee

1 Details on Limited-information Goodness-of-fit Testing

To avoid notation clutter, we will spell out the details under dichotomous responses and mention how the theory can be extended to polytomous data. Recall the $C = 2^n$-cell multinominal table on which the IRT model likelihood is define. We shall first write the $C \times 1$ vector of cell probabilities as $\boldsymbol{\pi}(\boldsymbol{\theta}) = [\pi_1(\boldsymbol{\theta}), \ldots, \pi_C(\boldsymbol{\theta})]'$. The true probabilities is given by $\boldsymbol{\pi}_0 = (\pi_{01}, \ldots, \pi_{0C})'$. Correspondingly, the $C \times 1$ vector of observed proportions is $\mathbf{p} = (p_1, \ldots, p_C)'$. Denote the $C \times 1$ vector of model-implied probabilities as $\hat{\boldsymbol{\pi}} = \boldsymbol{\pi}(\hat{\boldsymbol{\theta}}) = (\hat{\pi}_1, \ldots, \hat{\pi}_C)'$ after obtaining $\hat{\boldsymbol{\theta}}$ from the maximization of $L(\boldsymbol{\theta})$. The $C \times 1$ vector of cell residuals is given by $\mathbf{e} = \mathbf{p} - \hat{\boldsymbol{\pi}}$. Standard discrete multivariate analysis results suggest that $\mathbf{e}$ is asymptotically C-variate normally distributed under exactly correct model specification (see e.g., Bishop, Fienberg, & Holland, 1975)

$$\sqrt{N}\mathbf{e} = \sqrt{N}(\mathbf{p} - \hat{\boldsymbol{\pi}}) \xrightarrow{D} \mathcal{N}_C(\mathbf{0}, \boldsymbol{\Omega}), \quad (1)$$

where $\boldsymbol{\Omega} = \boldsymbol{\Xi} - \boldsymbol{\Delta}\mathcal{F}^{-1}\boldsymbol{\Delta}'$. The multinomial covariance matrix $\boldsymbol{\Xi}$ is equal to $\text{diag}(\boldsymbol{\pi}) - \boldsymbol{\pi}\boldsymbol{\pi}'$, and the Jacobian matrix is

$$\boldsymbol{\Delta} = \frac{\partial \boldsymbol{\pi}(\boldsymbol{\theta})}{\partial \boldsymbol{\theta}'}.$$

The Fisher information matrix is defined as $\mathcal{F} = \boldsymbol{\Delta}'[\text{diag}(\boldsymbol{\pi})]^{-1}\boldsymbol{\Delta}$.

In M_2, the marginal residuals up to order 2 are used. For n dichotomous items, there are n first-order marginal residuals, and $n(n-1)/2$ second-order marginal residuals. They are obtained via operator matrices. Let $\dot{\mathbf{L}}$ be an $n \times C$ fixed matrix consisting of zeros and ones. The (i, c)th element in $\dot{\mathbf{L}}$ is one if and only if item i is endorsed in the cth response pattern. Similarly, let $\ddot{\mathbf{L}}$ be an $n(n-1)/2 \times C$ fixed matrix of zeros and ones. Each row of this matrix corresponds to an item pair, and for each row, column c is equal to one if and only if the pair of items is endorsed in the cth response pattern.

Pre-multiplying $\mathbf{e}$ by $\dot{\mathbf{L}}$ leads to $\dot{\mathbf{e}} = \dot{\mathbf{L}}\mathbf{e}$, the $n \times 1$ vector of first-order marginal residuals. Similarly, pre-multiplying $\mathbf{e}$ by $\ddot{\mathbf{L}}$ leads to $\ddot{\mathbf{e}} = \ddot{\mathbf{L}}\mathbf{e}$, the $n(n-1)/2 \times 1$ vector of second-order marginal residuals. Stacking $\dot{\mathbf{e}}$ and $\ddot{\mathbf{e}}$, the vector of marginal residuals up to order 2 is $\mathbf{e}_2 = (\dot{\mathbf{e}}, \ddot{\mathbf{e}})$. Because $\mathbf{e}_2$ is a linear function of $\mathbf{e}$, the asymptotic distribution of $\mathbf{e}_2$ is also normal

$$\sqrt{N}\mathbf{e}_2 \stackrel{D}{\to} \mathcal{N}_{n(n+1)/2}(\mathbf{0}, \mathbf{L_2\Omega L_2'}), \tag{2}$$

where $\mathbf{L}_2 = (\dot{\mathbf{L}}, \ddot{\mathbf{L}})$, stacking $\dot{\mathbf{L}}$ and $\ddot{\mathbf{L}}$. Let us simplify the notation and let $\mathbf{\Omega}_2 = \mathbf{L}_2\mathbf{\Omega}\mathbf{L}_2' = \Xi_2 - \mathbf{\Delta}_2\mathcal{F}^{-1}\mathbf{\Delta}_2'$, where $\Xi_2 = \mathbf{L}_2\Xi\mathbf{L}_2'$ and $\mathbf{\Delta}_2 = \mathbf{L}_2\mathbf{\Delta}$. Specifically, $\mathbf{\Delta}_2$ is the matrix of derivatives of the first- and second order marginal probabilities with respect to $\boldsymbol{\theta}$.

Now Maydeu-Olivares and Joe's (2005) M_2 statistic can be defined:

$$M_2 = N\mathbf{e}_2' \left[\hat{\Xi}_2^{-1} - \hat{\Xi}_2^{-1}\hat{\mathbf{\Delta}}_2\left(\hat{\mathbf{\Delta}}_2'\hat{\Xi}_2^{-1}\hat{\mathbf{\Delta}}_2\right)^{-1}\hat{\mathbf{\Delta}}_2'\hat{\Xi}_2^{-1}\right]\mathbf{e}_2', \tag{3}$$

where $\hat{\mathbf{\Delta}}_2$ and $\hat{\Xi}_2$ denote the evaluation of $\mathbf{\Delta}_2$ and Ξ_2 at the maximum likelihood estimate $\hat{\boldsymbol{\theta}}$. It follows from Proposition 4 in Browne's (1984) that M_2 is asymptotically chi-square distributed with $n(n+1)/2 - \dim(\boldsymbol{\theta})$ degrees of freedom under the null hypothesis that the model fits *exactly* in the population.

For n polytomous items with K categories, the total number of possible response patterns becomes $C = K^n$. Consequently, the vectors of multinomial cell probabilities $\boldsymbol{\pi}(\boldsymbol{\theta})$ and $\hat{\boldsymbol{\pi}}$ is $C \times 1$ in size. In limited information goodness-of-fit testing, expanded operator matrices $\dot{\mathbf{L}}$ and $\ddot{\mathbf{L}}$ collapse cell probabilities into univariate and bivariate marginal probabilities or residual vectors, as detailed in Cai and Hansen (2013), among others. The asymptotic distribution theory of residuals under polytomous data can derived using along the same lines as Equation (2), enabling the formation of chi-square statistics similar to M_2 in Equation (3).

2 The Independence Model

Again, with no loss of generality, we illustrate the computations related to the full-independence model under dichotomous data. Recall that the 2PL model is

$$P(U_i = 1|\boldsymbol{\eta}) = \frac{1}{1 + \exp[-(\alpha + \boldsymbol{\beta}'\boldsymbol{\eta})]},$$

where α is the intercept term and $\boldsymbol{\beta}$ a potentially vector-valued item slope parameter conformable with the dimensions of $\boldsymbol{\eta}$. For the complete-independence null model, no latent variable is present

$$P(U_i = 1) = \frac{1}{1 + \exp(-\alpha)}. \quad (4)$$

Note that $P(U_i = 0) = 1 - P(U_i = 1)$.

Now let us turn to the computation of the M_2 statistic for the independence model. Requirement components are the first- and second-order marginal probabilities, the Jacobian, and the multinomial covariance matrix. As a practical matter, the fit statistics for independence model are implemented in flexMIRT®(Cai, 2015). We note that M_2 for independence model leads us to compute other relative fit indices such as the comparative fit index (CFI; Bentler, 1990), the normed-fit index (NFI; Bentler & Bonett, 1980), and the incremental fit index (IFI; Bollen, 1989) based on M_2.

2.1 Marginal probabilities

Let us begin with the computation of the first- and second-order marginal probabilities. Again, without loss of generality, we only consider dichotomous items. Let there be n items scored U_i = 0 or 1. Let $\dot{\pi}_i$ denote the first order marginal probability for item i and $\dot{\pi}_{ij}$ the second order marginal probability for item pair (i, j). Accordingly, $\dot{\pi}_i$ means the model-implied probability for item i when U_i = 1. Likewise, $\dot{\pi}_{ij}$ means the model-implied joint probability for items i and j when U_i = 1 and U_j = 1. We can compute $\dot{\pi}_i$

and $\dot{\pi}_{ij}$ directly as follows:

$$\dot{\pi}_i = \int P(U_i = 1|\boldsymbol{\theta})g(\boldsymbol{\eta})d\boldsymbol{\eta}, \tag{5}$$

$$\dot{\pi}_{ij} = \int P(U_i = 1|\boldsymbol{\theta})P(U_j = 1|\boldsymbol{\theta})g(\boldsymbol{\eta})d\boldsymbol{\eta}. \tag{6}$$

In our independence model, the first-order marginal probability when $U_i = 1$ becomes

$$\dot{\pi}_i = \frac{1}{1+\exp(-\alpha)} \int g(\boldsymbol{\eta})d\boldsymbol{\eta} = \frac{1}{1+\exp(-\alpha)}. \tag{7}$$

It follows that $\dot{\pi}_i = \dot{p}_i$, where $\dot{p}_i$ is the observed counterpart. This is due to the fact that intercepts perfectly fit the observed univariate proportions. Once this is established, the second-order marginal probabilities can be obtained simply as the products of two observed univariate proportions, i.e., $\dot{\pi}_{ij} = \dot{p}_i\dot{p}_j$.

2.2 The Jacobian

Next, the Jacobian, Δ_2, for the independence model can be expressed as

$$\boldsymbol{\Delta}_2 = \frac{\partial \boldsymbol{\pi}(\hat{\boldsymbol{\theta}})}{\partial \boldsymbol{\theta}'} = \frac{\partial \boldsymbol{\pi}(\hat{\boldsymbol{\alpha}})}{\partial \boldsymbol{\alpha}'} = \begin{pmatrix} \frac{\partial \dot{\pi}(\hat{\boldsymbol{\alpha}})}{\partial \boldsymbol{\alpha}'} \\ \frac{\partial \ddot{\pi}(\boldsymbol{\alpha})}{\partial \boldsymbol{\alpha}'} \end{pmatrix}.$$

Note that we only have to take the first-order derivatives of the model-implied marginal probabilities with respect to intercept. A typical elements in the upper block is

$$\frac{\partial \dot{\pi}_i}{\partial \alpha_i} = \frac{\partial P(U_i = 1)}{\partial \alpha_i} = \dot{\pi}_i(1 - \dot{\pi}_i), \tag{8}$$

and those of the lower block are

$$\begin{aligned} \frac{\partial \ddot{\pi}_{ij}}{\partial \alpha_i} &= \frac{\partial P(U_i = 1)}{\partial \alpha_i} P(U_j = 1) = \dot{\pi}_i(1 - \dot{\pi}_i)\dot{\pi}_j \\ \frac{\partial \ddot{\pi}_{ij}}{\partial \alpha_j} &= \frac{\partial P(U_j = 1)}{\partial \alpha_j} P(U_i = 1) = \dot{\pi}_j(1 - \dot{\pi}_j)\dot{\pi}_i. \end{aligned} \tag{9}$$

2.3 The multinomial covariance matrix

The multinominal covariance matrix Ξ_2 may be rewritten as (see Cai & Hansen, 2013):

$$\Xi_2 = \boldsymbol{\Sigma} - \boldsymbol{\pi}_2\boldsymbol{\pi}_2', \tag{10}$$

where $\boldsymbol{\Sigma} = \mathbf{L}_2\text{diag}(\boldsymbol{\pi})\mathbf{L}_2'$, and can be partitioned into $\boldsymbol{\Sigma}_{11}$, $\boldsymbol{\Sigma}_{21}$, and $\boldsymbol{\Sigma}_{22}$ as follows:

$$\boldsymbol{\Sigma} = \begin{pmatrix} \boldsymbol{\Sigma}_{11} & \\ \boldsymbol{\Sigma}_{21} & \boldsymbol{\Sigma}_{22} \end{pmatrix} = \begin{pmatrix} \dot{\mathbf{L}}\text{diag}(\boldsymbol{\pi})\dot{\mathbf{L}}' & \\ \ddot{\mathbf{L}}\text{diag}(\boldsymbol{\pi})\dot{\mathbf{L}}' & \ddot{\mathbf{L}}\text{diag}(\boldsymbol{\pi})\ddot{\mathbf{L}}' \end{pmatrix} \tag{11}$$

In short, calculating the elements of $\boldsymbol{\Sigma}$ involves calculation of the first, second, third, and fourth order marginal probabilities. Because of full-independence, the model-implied probabilities for the third and fourth order margins can be obtained as products of univariate proportions.

References

Bentler, P. M. (1990). Comparative fit indices in structural models. *Psychological Bulletin*, *107*, 238-–246. doi: 10.1037/0033-2909.107.2.238

Bentler, P. M., & Bonett, D. G. (1980). Significance tests and goodness of fit in the analysis of covariance structures. *Psychological Bulletin*, *88*, 588-–606. doi: 10.1037/0033-2909.88.3.588

Bishop, Y. M. M., Fienberg, S. E., & Holland, P. W. (1975). *Discrete multivariate analysis: Theory and practice*. Cambridge, MA: MIT Press.

Bollen, K. A. (1989). A new incremental fit index for general structural equation models. *Sociological Methods & Research*, *17*(3), 300–316.

Browne, M. W. (1984). Asymptotically distribution-free methods for the analysis of covariance structures. *British Journal of Mathematical and Statistical Psychology*, *37*, 62–83. doi: 10.1111/j.2044-8317.1984.tb00789.x

Cai, L. (2015). *flexMIRT® Version 3.0: Flexible multilevel multidimensional item analysis and test scoring.* Chapel Hill, NC: Vector Psychometric Group, LLC.

Cai, L., & Hansen, M. (2013). Limited-information goodness-of-fit testing of hierarchical item factor models. *British Journal of Mathematical and Statistical Psychology*, *66*, 245-276. doi: 10.1111/j.2044-8317.2012.02050.x

Maydeu-Olivares, A., & Joe, H. (2005). Limited- and full- information estimation and testing in 2^n contingency tables: A unified framework. *Journal of the American Statistical Association*, *100*, 1009–1020. doi: 10.1198/016214504000002069
